# Preparation of TiO_2_/Black Talc Composite Photocatalyst and the Research on Its Adsorption-Degradation Coupling Effects

**DOI:** 10.3390/ma14206038

**Published:** 2021-10-13

**Authors:** Huan Shuai, Yuxin Wang, Jiao Wang, Gaoxiang Du, Daimei Chen, Yu Liang

**Affiliations:** 1 School of Materials Science and Technology, China University of Geosciences, Beijing 100083, China; shuaihuan@email.cugb.edu.cn (H.S.); 2103190011@email.cugb.edu.cn (Y.W.); 2 Beijing Yiyi Star Technology Co., Ltd., Beijing 100089, China; 3 School of Basic Education, Beijing Polytechnic College, Beijing 100042, China; 4 School of Materials Science and Technology, Shenyang University of Chemical Technology, Shenyang 110142, China; liangyu1234@syuct.edu.cn

**Keywords:** nano-TiO_2_ particles, black talc, characteristic adsorption, photodegradation, synergistic effect

## Abstract

In this paper, a TiO_2_/black talc composite photocatalyst was prepared by the sol-gel method using TBOT as titanium source and black talc as carrier. Rhodamine B was used as the targeted pollutant to study the adsorption role of carbon in black talc. The results showed that with the adsorption-degradation cycles, the illumination time can be reduced by 40%. The adsorption rate and degradation rate of the composite photocatalyst was also increased. The degradation rate of Rhodamine B reached more than 95%, which fully shows the synergistic effect between TiO_2_ nanoparticles and black talc. In this way, the adsorption-degradation coupling of the photocatalyst could be realized.

## 1. Introduction

With the development of industry in recent years, organic pollutants in air and water are emerging and increasing with astonishing speed, causing a series of environmental issues [1,2] and health problems [3] that affect our lives seriously. Therefore, it is very important to investigate an environmentally friendly, low-cost, simple, facile, and efficient method [4] to deal with the above-mentioned problem. Among the various solutions, physical adsorption [5,6] and photocatalytic degradation [7,8,9,10,11] are regarded as two common, effective solutions for pollution treatment. Titanium dioxide (TiO_2_) has been well known as a star photocatalyst for organic pollution in air and water for its superiority of low price, stable chemical properties [12,13,14,15,16], suitable conduction band and valence band potential, no secondary pollution, and low photocorrosion [17,18,19]. However, TiO_2_ has some disadvantages, such as low specific surface area, high photogenerated electron-hole recombination rate, narrow light utilization wavelength range, high agglomeration rate, and difficulty to be recycled [20], which limit its further applications. Therefore, it is urgent to find a carrier with porous structure and stable properties to load TiO_2_ to overcome the above-mentioned problems [21].

Non-metallic minerals, such as diatomite, zeolite, and sepiolite, have been widely used as carriers [22,23] due to their low cost, excellent stability, and large specific area. Biochar, such as biomass bamboo fiber [24], macroalgae [25], coconut shell biochar [26], microalgae, and nut shells [27], has also been shown to promote the catalytic effect of photocatalysts and enhance the adsorption of organic molecules [28]. Pinna et al. [27] produced a biochar-decorated TiO_2_ photocatalyst through a simple drop casting method. The composite Biochar -TiO_2_ material has a better catalytic effect than pure titanium dioxide, which proves that BC NPs have the ability to act as a promoter. Moreover, the enhanced adsorption of organic molecules, coupled with the improved charge carrier separation provided by BC NPs, resulted in a nearly two-fold increase in photocatalytic performance. Zhang et al. [29] synthesized TiO_2_ supported acid activated sepiolite (TiO_2_/AAS) fibers under low temperature conditions and it was found that the specific surface area of the composite material was much larger than that of the original sepiolite. The photocatalytic activity of the composite was also excellent and superior to that of the TiO_2_/sepiolite (raw sepiolite) and pure TiO_2_. The addition of sepiolite significantly improved the photocatalytic activity of TiO_2_. Suá et al. added TiO_2_ to zeolite and found that the photocatalytic activity of the composite was ten times better than that of the pure TiO_2_ particles. The more uniform the morphology of TiO_2_ particles, the better the oxidation ability [30]. Liu et al. [31] prepared TiO_2_/zeolite composite material by the sol-gel method and studied its adsorption and photocatalytic degradation performance for sulfadiazine (SDZ) under ultraviolet light irradiation. The results showed that, under neutral pH value, 90% of SDZ can be removed by TiO_2_/zeolite within 120 min. Black talc is a kind of non-metallic mineral with excellent surface affinity, chemical stability, and thermal stability [32]. Compared with other minerals, it has a unique carbon layer between the black talc layers and the crystal grains of talc, which is beneficial to gather the surrounding organic pollutants, thus improving the catalytic efficiency of the photocatalyst.

In this study, a TiO_2_/black talc composite photocatalyst was prepared by the sol-gel method. Using black talc as carrier can not only increase the contact area, but also facilitate the dispersion of TiO_2_, resulting in the improvement of the photodegradation efficiency of the photocatalyst. The innovation of this research lies in the use of the adsorption degradation cycle test method, which can make full use of the adsorption properties of black talc and the degradation performance of TiO_2_ in the composite photocatalyst, thus improving the overall rate of adsorption degradation. Compared with traditional degradation method, it can reduce the light time by 60% and reduce the energy consumption. This study is of great significance for the comprehensive utilization of black talc.

## 2. Materials and Methods

### 2.1. Materials

Black talc was purchased from Guangfeng District, Jiangxi, China. Tetrabutyl titanate (TBOT) was purchased from Aladdin Reagent Co., Ltd. (Shanghai, China). The sulfuric acid, sodium hydroxide, and analytical pure ethanol were purchased from Beijing Chemical Plant (Beijing, China)). Deionized water was used throughout the experiment.

### 2.2. Synthesis of the Composite Material

In a typical synthesis, excess 10% sulfuric acid solution was added to 300 mesh black talc powder and the suspension was stirred for 2 h. Then, the mixture was filtered and washed with DI H_2_O for three times. Finally, it was dried at 105 °C for 24 h prior to use and was labelled as BT (black talc). BT-OC (Oxygen-calcined black talc) was prepared by the calcination of BT sample at 550 °C for 2 h in air. The composite photocatalyst was prepared as follows. A certain amount of TBOT was dissolved in ethanol. Then, BT was added, and the mixture was stirred for one hour and dried at 105 °C. Then, it was calcined at 550 °C for 2 h under N_2_ atmosphere. The obtained composite photocatalyst was labelled as BT-xT, where x is the mass ratio of TiO_2_ to black talc.

### 2.3. Photodegradation Experiment

Photocatalytic activities of samples were evaluated by their performance as catalysts in the photocatalytic oxidation of RhB in water. In this experiment, 1.5 mg photocatalyst was dispersed in 50 mL of RhB solution (30 mg/L). As a control group, 50 mL of RhB solution (30 mg/L) was taken without adding anything. Prior to degradation, the suspension was magnetically stirred in the dark for a period of time to establish adsorption–desorption equilibrium between the pre-irradiation photocatalyst and RhB. High-pressure mercury lamp with a power of 300 W was chosen as the light source of photodegradation and its dominant wavelength was 365 nm. At given intervals of illumination, a specimen (3 mL of the suspension was collected and centrifuged. The filtrates were analyzed by UV-vis spectroscopy at 554 nm.

### 2.4. Characterization Methods

X-ray diffraction (XRD) patterns of the samples were recorded using a Bruker D8 Advance diffractometer (Bruker, Germany), with CuKαradiation (λ = 0.15418) at 40 kV and 30 mA. Scanning electron microscopy (SEM) (JSM7500F, JEOL, Tokyo, Japan) was used to observe the microstructure of the samples. X-ray photoelectron spectra (XPS) were obtained using the radiation of Al Kα line (1486.6 eV, 300 W) as the excitation source. Binding energies were referenced to the C1s peak at 284.8 eV. The BET surface area of the samples was determined by N_2_ adsorption by using NOVA4000 equipment (Quantachrome, Boynton Beach, FL, USA). Prior to N2 adsorption, the samples were evacuated at 473 K under vacuum for 4 h.

## 3. Results

### 3.1. The Structure, Morphologies and Composition of the Prepared Catalysts

**Figure 1:**
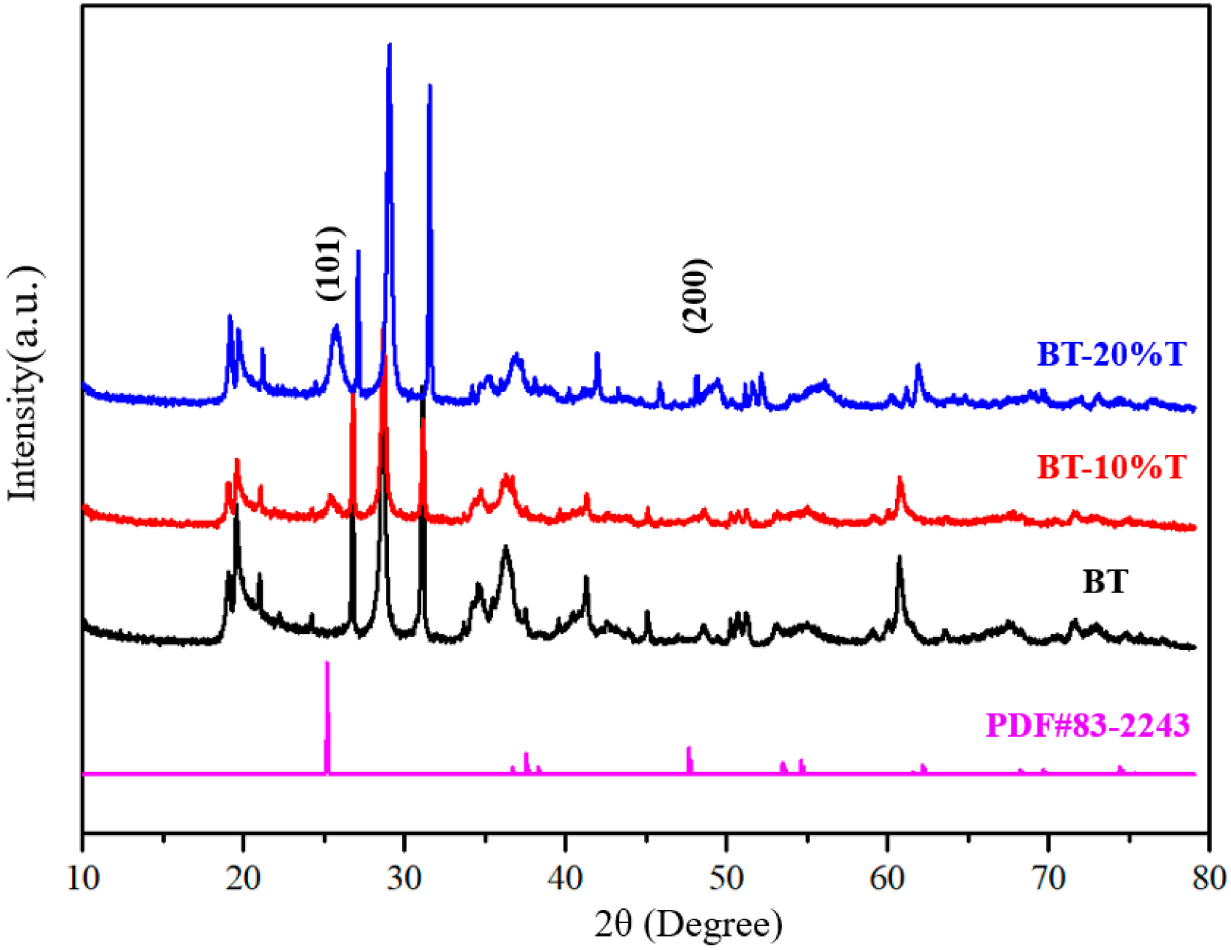
XRD diffraction patterns for photocatalytic material.

X-ray diffraction analysis was used to analyze the phase structure and composition of the prepared samples. Figure 1 shows the XRD patterns for BT, BT-10%T, and BT-20%T samples. The characteristic diffraction peaks for the anatase phase are observed in both BT-10%T and BT-20%T samples, suggesting that the anatase phase of the TiO_2_ has been successfully synthesized on black talc [33]. The intensities of the characteristic peaks of talc decreased with the TBOT amount and all the characteristic peaks of talc are observed in the composite photocatalyst, which indicates that calcination at 550 °C cannot damage the black talc structure.

**Figure 2:**
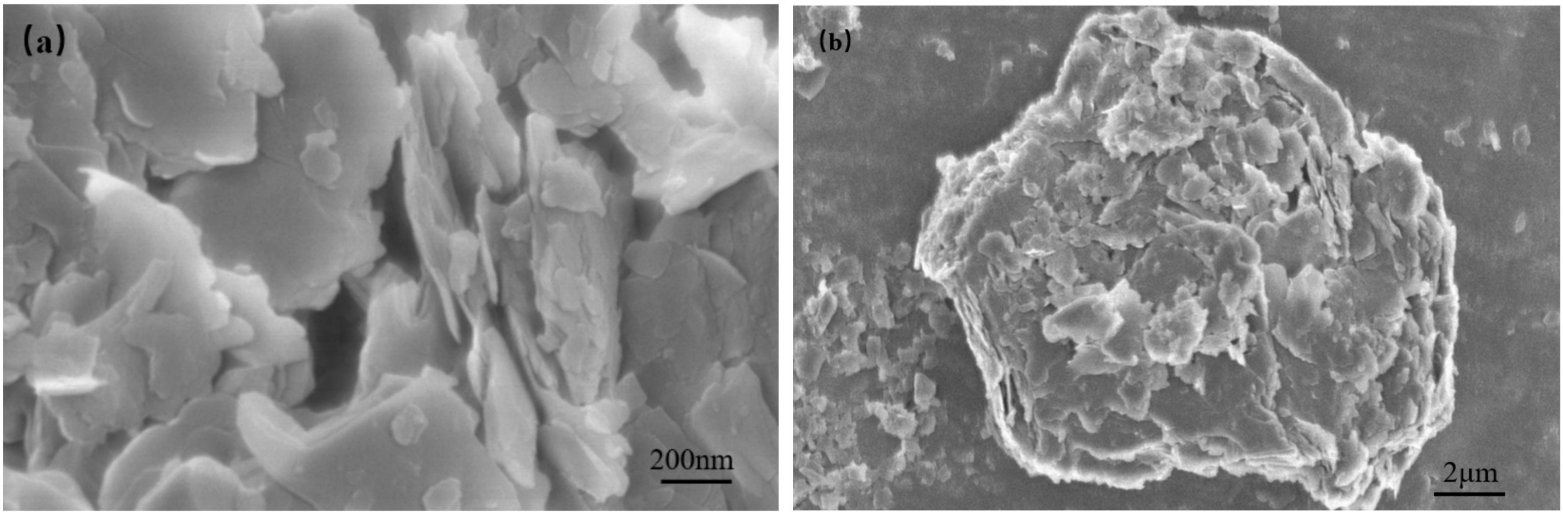

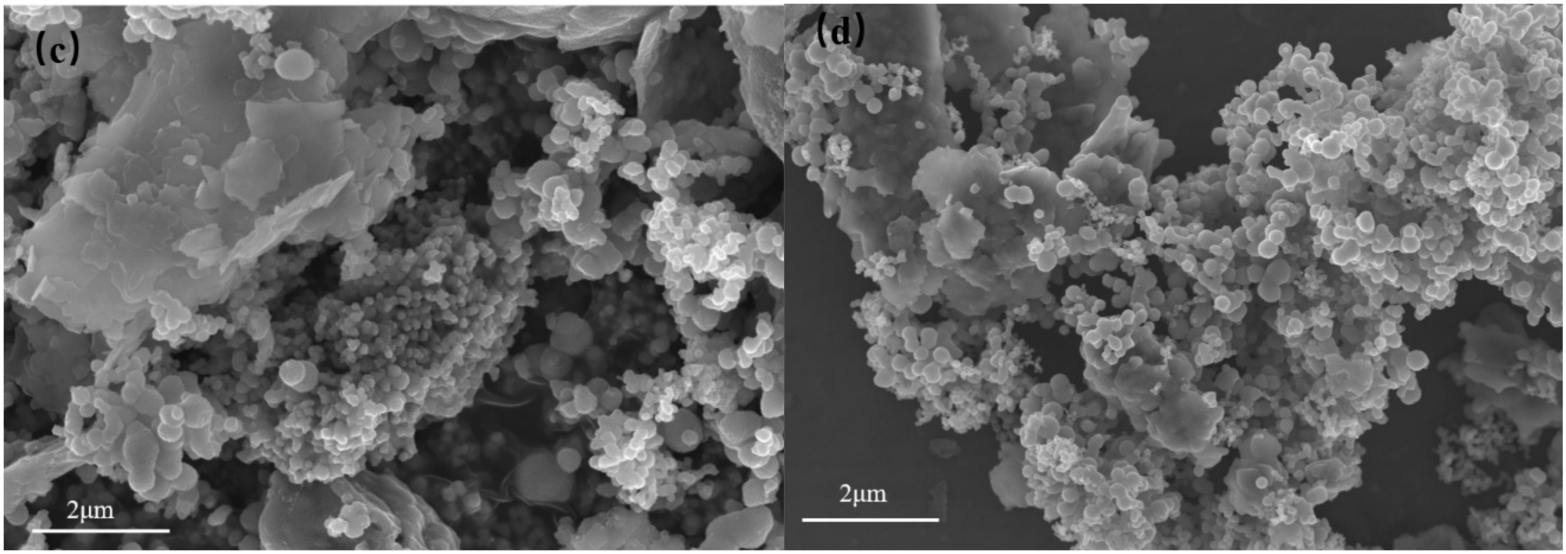
(**a**,**b**) SEM images of black talc; (**c**,**d**) SEM images of the photocatalyst.

Figure 2 shows SEM images of black talc and BT samples. It can be seen that black talc exhibits a layered structure with different sizes (Figure 2a,b). Most of the particle sizes are less than 2 microns, but a minority can reach hundreds of microns. In the prepared photocatalyst composite, a large amount of TiO_2_ particles with uniform size of 50 nm are agglomerated on the surface of the black talc (Figure 2c,d).

**Figure 3:**
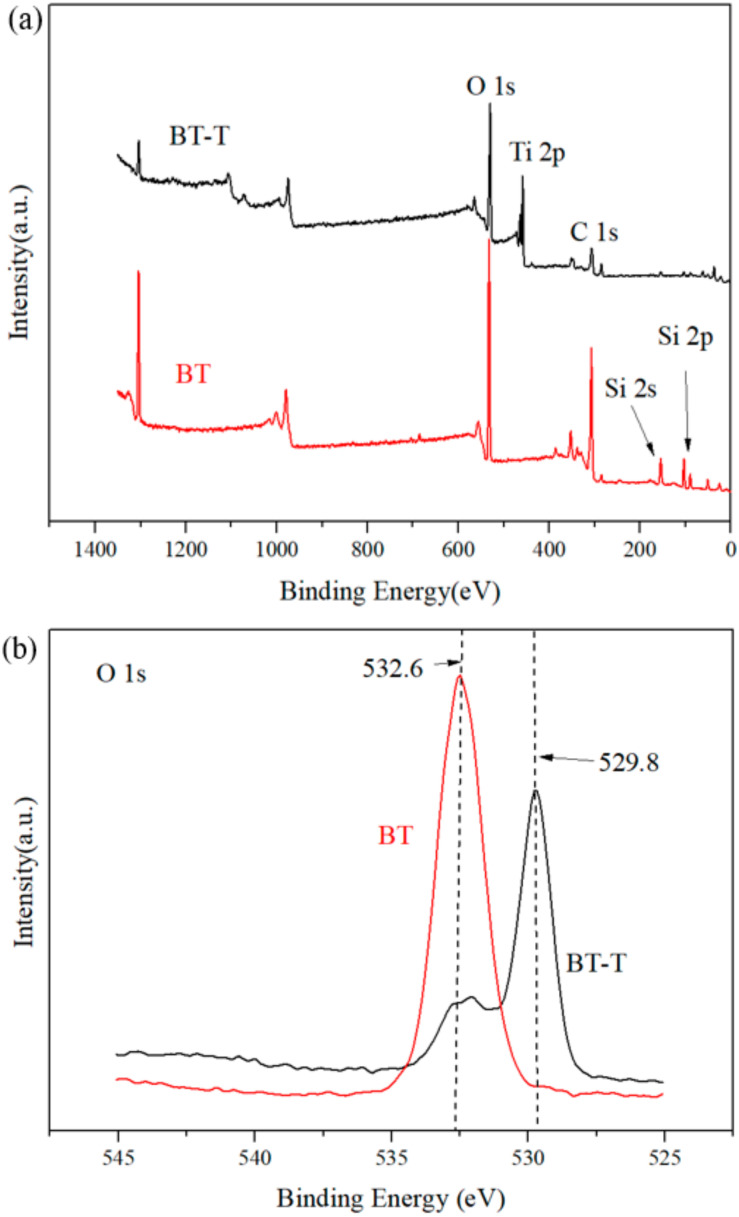
(**a**) XPS survey spectra of BT and BT-T, (**b**) O 1s high-resolution energy spectrum.

In order to further analyze the interaction between the TiO_2_ and black talc, X-ray photoelectron spectroscopy (XPS) was used to test and analyze BT and BT-T (Figure 3). Figure 3a shows that BT-T has a characteristic peak of Ti 2p, which is not existed in BT. This indicated that TiO_2_ was successfully loaded on black talc. The analysis of the chemical state of O 1s is shown in Figure 3b. There are two characteristic peaks emerging at 532.6 eV and 529.8 eV for BT-T, which can be attributed to the existence of Si-O-Si and Ti-O-Si, respectively [34,35], while only one characteristic peak can be observed in BT sample. From this comparison, it can be seen that there is a chemical bonding between TiO_2_ and black talc, which indicates the good stability of the composite.

### 3.2. Nitrogen Adsorption-Desorption Isotherms of the Prepared Catalysts

**Figure 4:**
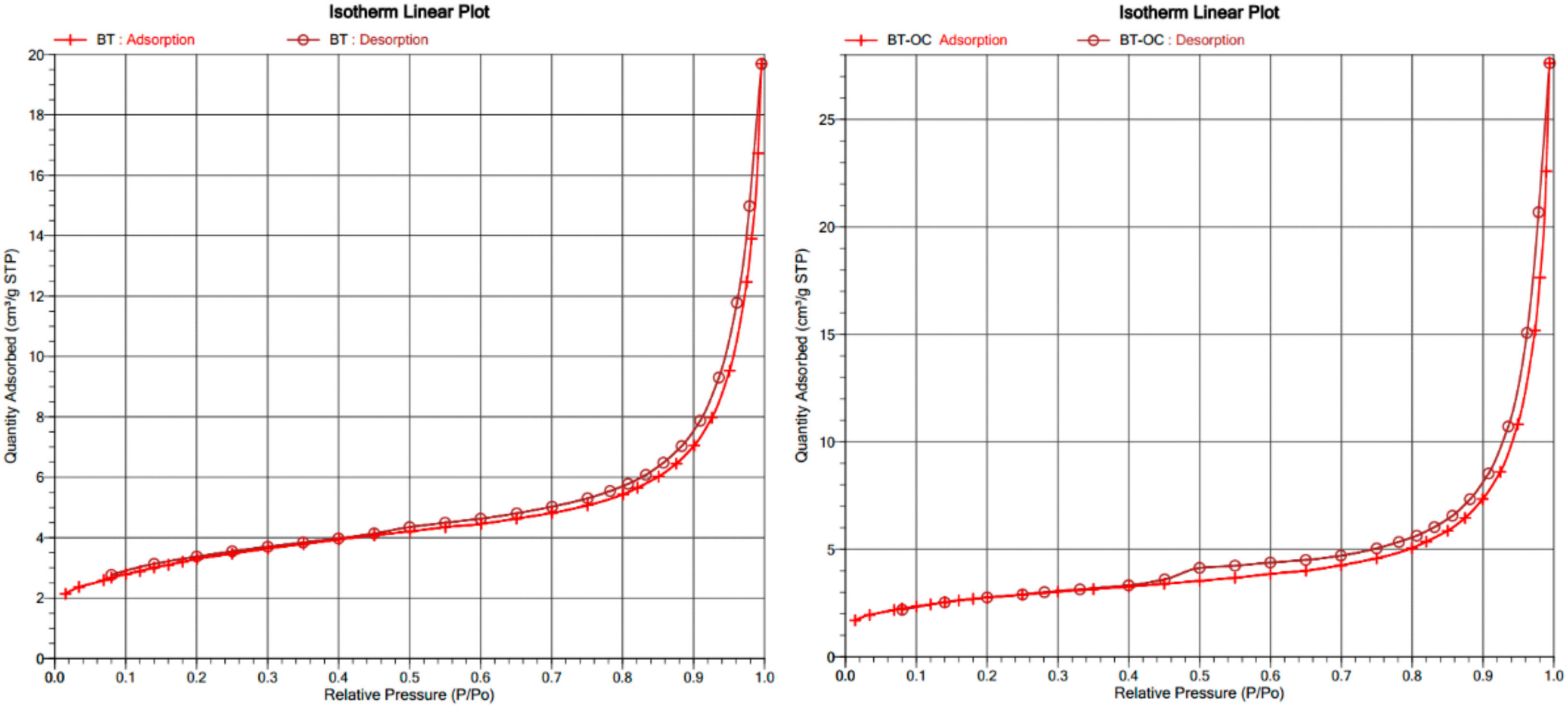
N_2_ adsorption-desorption isotherms of BT and BT-OC samples.

N_2_ adsorption-desorption isotherms of black talc and calcined talc are presented in Figure 4. Both the black talc and calcined talc have almost the same value of specific surface area, i.e., 11.96 m^2^/g and 10.17 m^2^/g, respectively. This means that the pore structure of talc is not affected by the calcination at 550 °C, which is consistent with the analysis of XRD results. Since both the nitrogen adsorption and desorption curves of the two samples have hysteresis loops, which fit in typical Ⅳ according to the classification of the International Union of Pure Theory and Applied Chemistry (IUPAC), it can be seen that both BT and BT-OC samples have mesoporous structures.

### 3.3. The Adsorption Behavior and Photodegradation Behavior of the Prepared Photocatalysts

**Figure 5:**
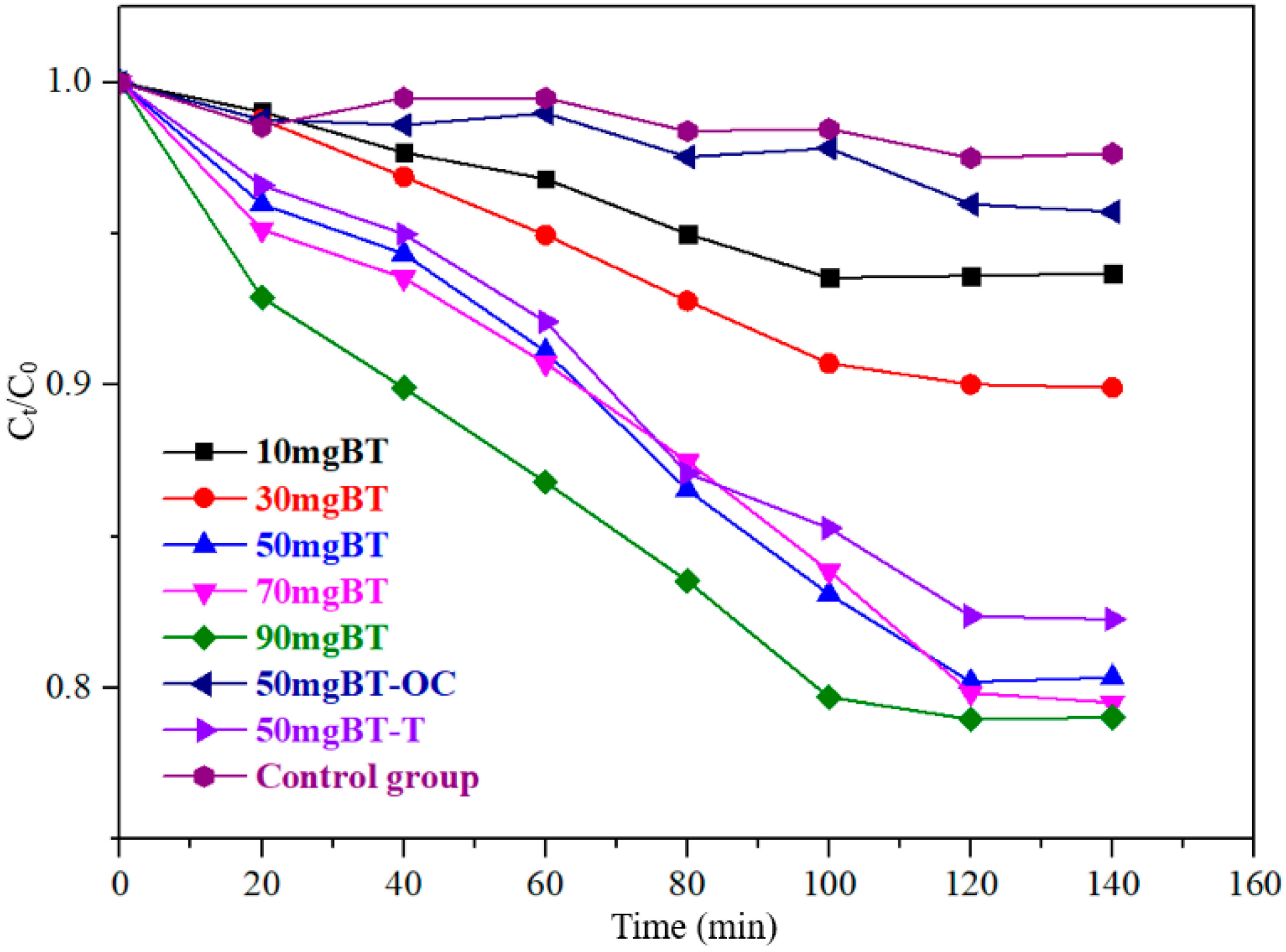
The adsorption effect curves of RhB on different samples.

Figure 5 shows the adsorption effect curves of different samples for Rhodamine B. Under dark conditions, the compound Rhodamine B itself in control group is relatively stable with only 2.4% decay in the concentration. The adsorption rate of Rhodamine B by uncalcined black talc is gradually improved from 5.3% to 21.0% with the increase of dose amount from 10 mg to 90 mg. However, compared with BT sample (50 mg), the adsorption rate of Rhodamine B by BT-OC calcined by oxygen decreased sharply to 4.3%, which was only a little higher than the control group. This indicated that oxygen calcination could remove organic carbon that can be used to adsorb Rhodamine B. It also shows that the adsorption rate of BT-T (50 mg) was 17.8%, which was slightly lower than the adsorption rate of 50 mg BT (19.7%). This is caused by the adhesion of TiO_2_ on the surfaces of black talc, which can affect part of the role of carbon, resulting in a decrease in adsorption rate. After 120 min, the adsorption rate remained basically unchanged, meaning that the absorption equilibrium was achieved.

**Figure 6:**
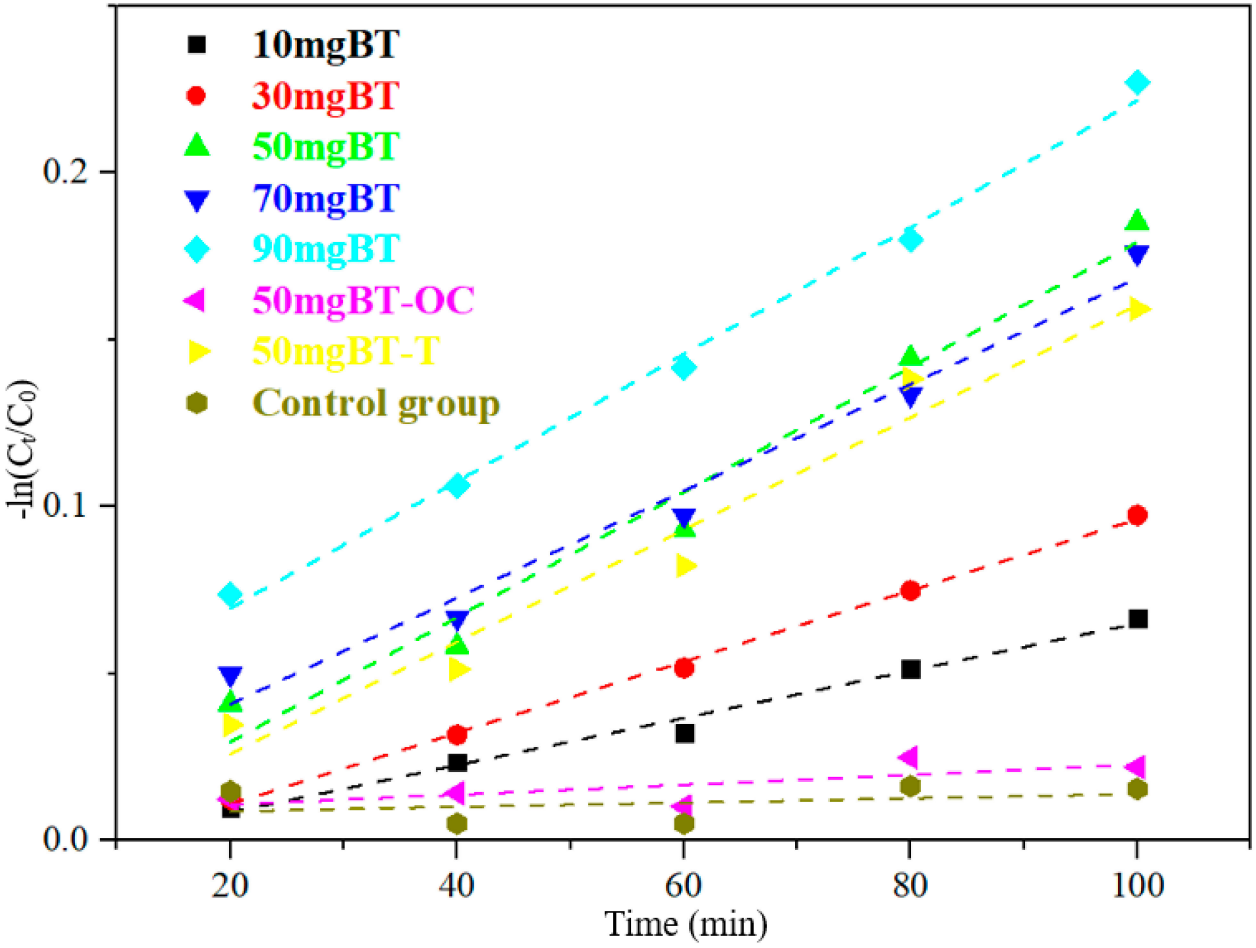
Kinetics fitting curves for the adsorption of RhB.

The physical adsorption of Rhodamine B solution to black talc follows the quasi-first-order kinetic equation, as shown in Figure 6, where the adsorption performance for each sample can be quantitatively evaluated through the apparent rate constant k.

−ln(C/C_0_) = kt (1)

Table 1 below shows the value of the correlation coefficient R^2^ of the kinetic equation and the apparent rate constant k. It can be seen that the correlation coefficient of the kinetic equation of BT-OC and the control group is not high. This is due to the small adsorption capacity of these two groups. The test data fluctuate greatly, causing the R^2^ value to be small, and the correlation coefficients of the other groups of kinetic equations are higher, which can be fitted by the kinetic equation. The maximum value of the apparent rate constants 50 mg BT and 90 mg BT is 0.0019, which shows that the adsorption rate of 50 mg BT and 90 mg BT is the fastest, followed by 50 mg BT-T. In order to maximize the reaction efficiency, 50 mg sample was used in the degradation stage of this experiment, which had the highest adsorption efficiency.

After the adsorption experiment, the photocatalytic degradation experiment was conducted on the BT and BT-T samples. At this stage, a set of adsorption degradation cycle groups was added to compare with constant light degradation experiments. The cycle process contained 20 min degradation followed by 20 min adsorption and it would be continued until rhodamine B was completely degraded.

**Figure 7:**
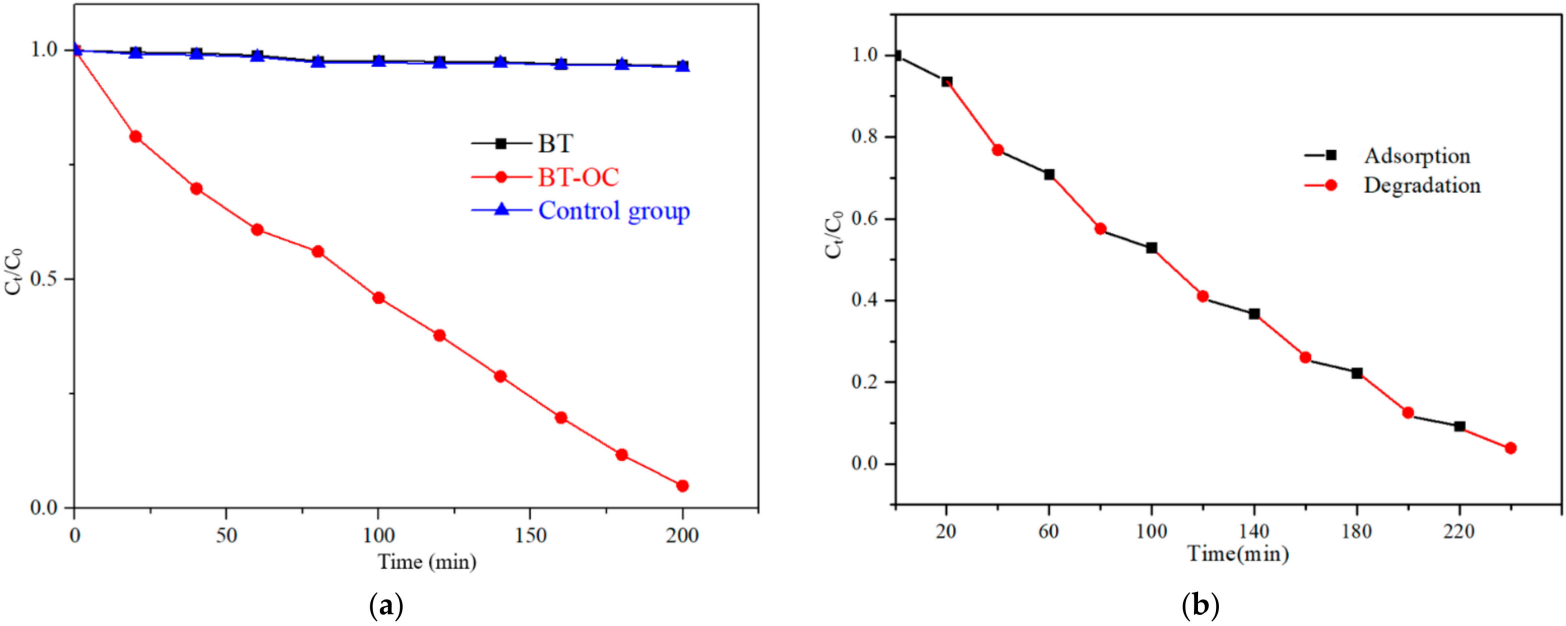
(**a**) Degradation effect curves of RhB with the different samplesunder UV irradiation. (**b**) The adsorption-degradation effect curve of RhB with BT-T.

Figure 7 shows the degradation effect curves of different samples on RhB. Under UV irradiation, the concentration of RhB in BT and the control group changed slightly (both less than 4%), indicating that Rhodamine B was quite stable under UV irradiation. The degradation rate of BT-T after 200 min irradiation reached 95.1% and the degradation rate of BT-T in the adsorption-degradation cycle group was 96.2%. Although the results were very similar, the total irradiation time of BT-T in the adsorption-degradation cycle group was 120 min, which was only 60% of the total irradiation time in the continuous light group, thus proving the excellent effect of the adsorption degradation cycle groups. The degradation cycle method can greatly reduce the use of light and achieve a degradation efficiency slightly higher than that of constant lightening.

In order to compare the coupling performance of BT-T and BT-T cycle on the adsorption and degradation of Rhodamine B, we performed the kinetic fitting of the adsorption and degradation of Rhodamine B for 120 min.

**Figure 8:**
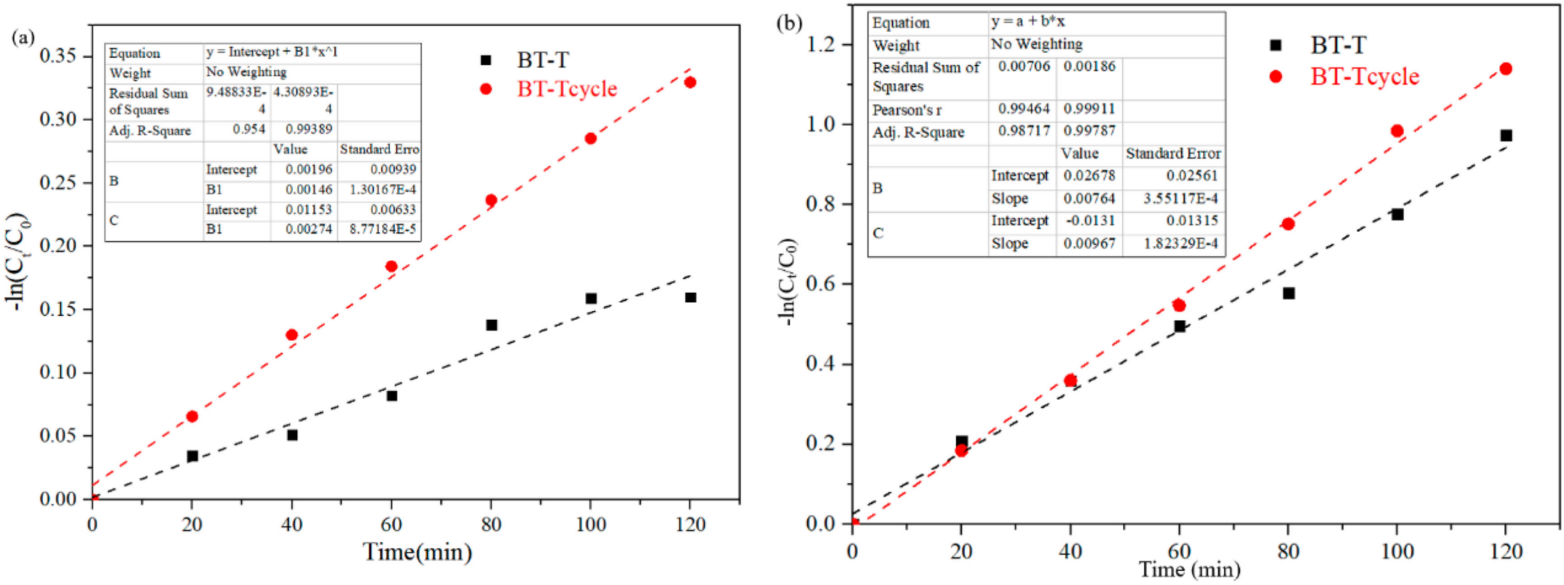
(**a**) Kinetics fitting plots for the adsorption of RhB. (**b**) Kinetics fitting plots for the degradation of RhB.

As shown in Figure 8, the apparent rate constants of adsorption and degradation of BT-T cycle are 0.00274 and 0.00967, respectively. The apparent rate constants of adsorption and degradation of BT-T are 0.00146 and 0.00764, respectively. The adsorption and degradation rates of the former are respectively 1.88 times and 1.27 times of the latter, showing that the adsorption and degradation recycling can increase the rate of adsorption and degradation and reduce the time required for the reaction. This is because in the stage of pure adsorption of BT-T, the adsorption capacity will reach saturation. After the pores are full, the adsorption capacity will be significantly weakened, but the cyclic method can degrade the adsorbed RhB in time, making the adsorption capacity decrease to a small extent.

**Figure 9:**
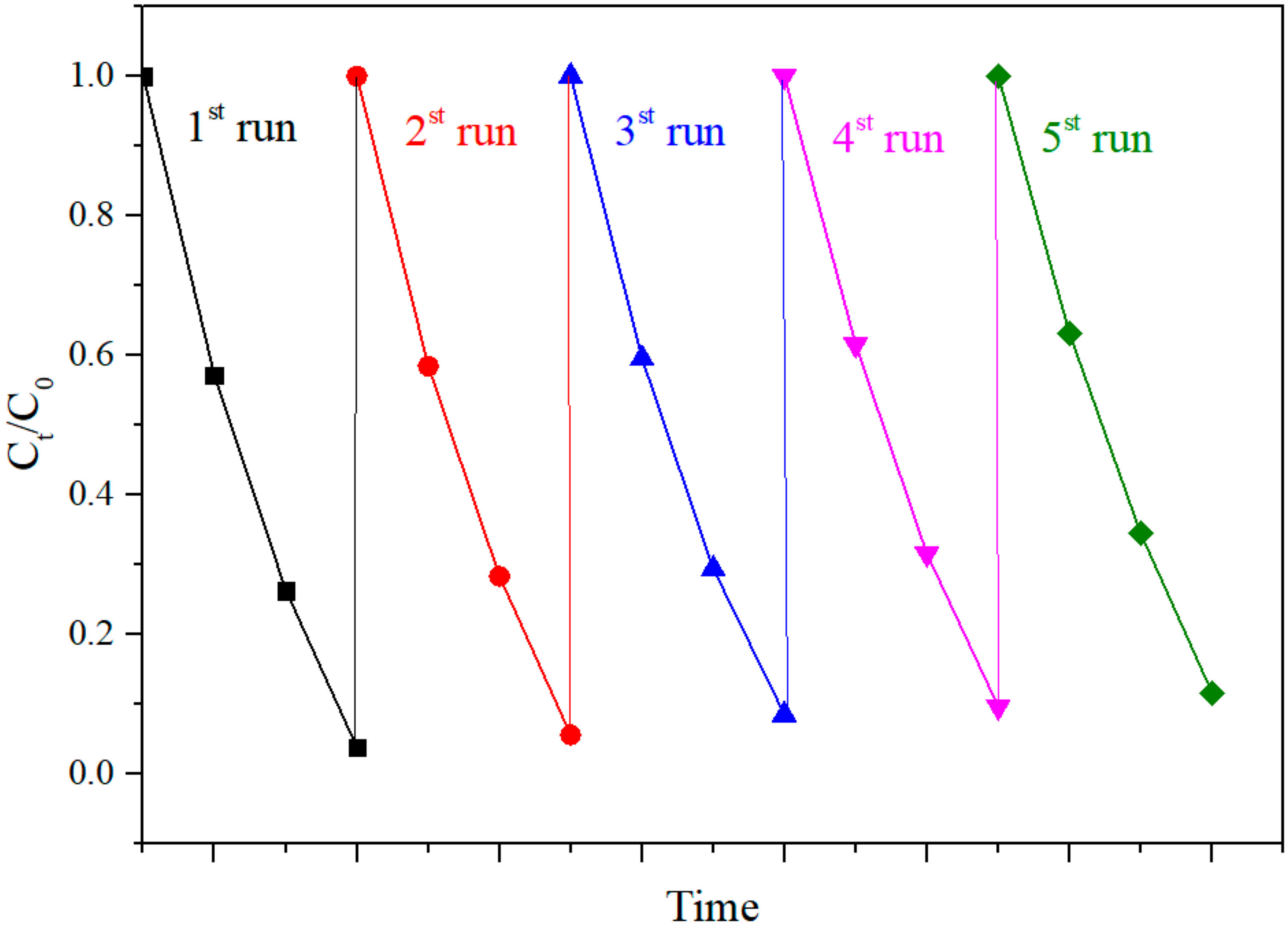
Cycling degradation test of RhB on BT-T sample.

In order to further test the stability of the prepared photocatalyst, cyclic degradation tests were conducted, and the results are shown in Figure 9. The experimental results show that the adsorption-degradation rate for RhB being catalyzed by BT-T was still around 90% after five cycles, indicating good stability and reusability.

## 4. Discussion

**Scheme 1:**
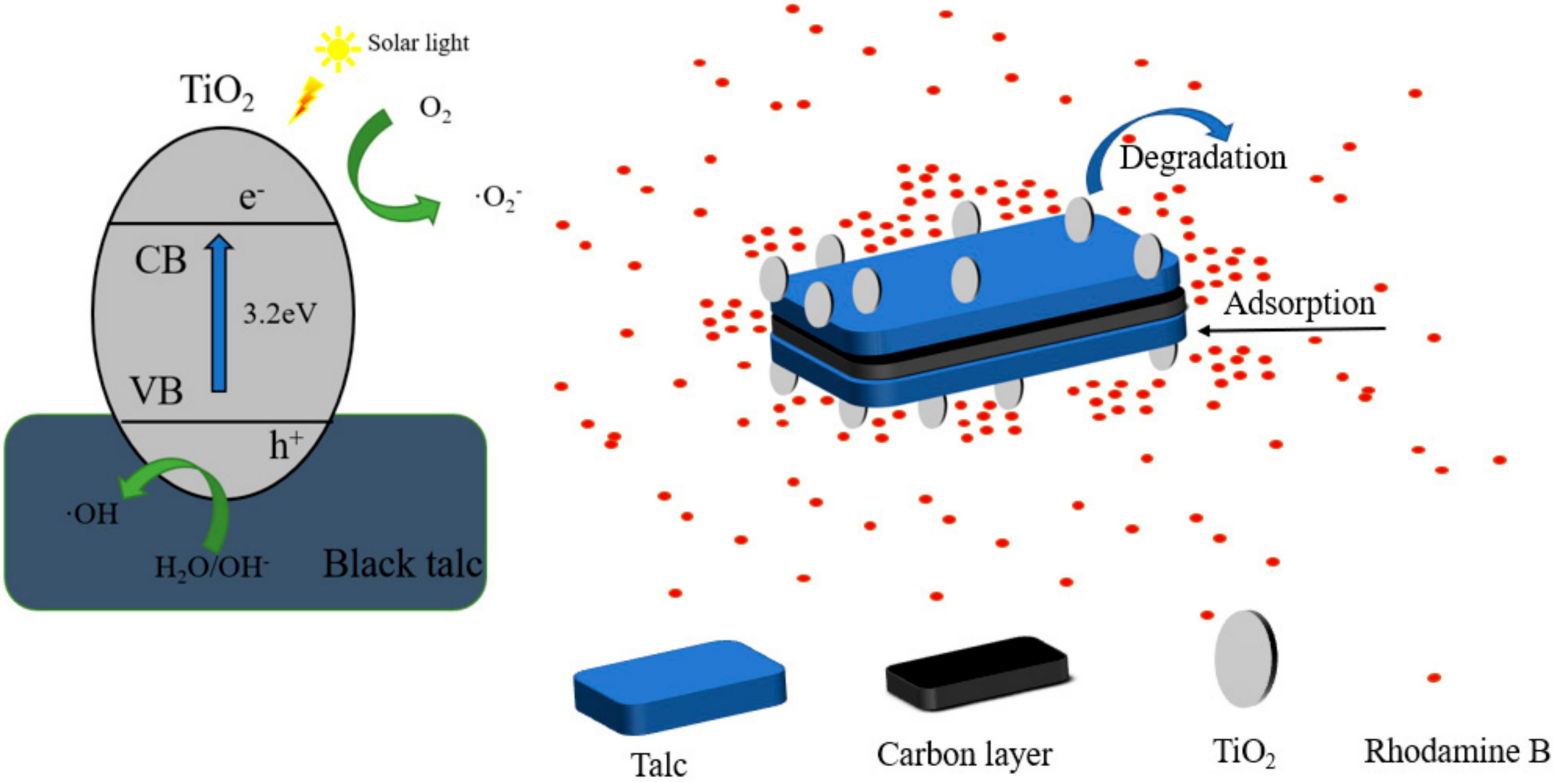
Schematic diagram of photocatalytic performance enhancement mechanism of BT-T composite material.

Based on the above analysis, the photocatalytic mechanism of BT-T composite was proposed. As shown in Scheme 1, TiO_2_ nanoparticles adhered to the surfaces of black talc and formed stable Si-O-Ti bonds. This can avoid agglomeration and ensure that most of TiO_2_ can be exposed to contact with pollutants. Moreover, there is a graphene-like carbon layer in black talc, which has a characteristic adsorption effect on organic pollutants. Thus, organic pollutants can be gathered around black talc. TiO_2_ nanoparticles that were adhered on the surfaces of black talc can degrade pollutants and reduce the concentration of the pollutants, which in turn can promote the adsorption of the pollutants. Therefore, both TiO_2_ and black talc have synergic effects on each other, thus improving the photocatalytic activity of the prepared composite.

## 5. Conclusions

In this study, a TiO_2_/black talc photocatalyst was prepared through the sol-gel method. Black talc combined with TiO_2_ via chemical bonds and the composite demonstrated good stability. The adsorption-degradation cycle can significantly improve the coupling performance of the photocatalyst for rhodamine B. Compared with the constant light degradation group, it can reduce irradiation time by 60% and reduce energy consumption. The degradation rate of Rhodamine B by the composite photocatalyst could reach more than 95% after 120 min and the degradation rate of the composite photocatalyst was more than 90% after five cycles. Black talc can adsorb and enrich the pollutants around the photocatalyst, which helps to improve its catalytic efficiency. Therefore, black talc can be used as an effective carrier for the improvement of semiconductor photocatalyst performance and cost reduction.

## Figures and Tables

**Table 1:** The value of the correlation coefficient R^2^ and constant k of the kinetic equation.

Time	10 mgBT	30 mgBT	50 mgBT	70 mgBT	90 mgBT	50 mgBT-OC	50 mgBT-T	Control Group
R^2^	0.98873	0.99967	0.98733	0.99865	0.99944	0.17109	0.94654	0.1593
K	0.0007	0.0011	0.0019	0.0016	0.0019	0.0001	0.0017	0.00006

## Data Availability

The data presented in this study are available on request from the corresponding author.
